# Anoplocephalid tapeworms in mountain gorillas (*Gorilla beringei beringei*) inhabiting the Volcanoes National Park, Rwanda

**DOI:** 10.1017/S0031182023001178

**Published:** 2024-02

**Authors:** Barbora Červená, Tereza Prokopová, Rita Maria Cameira, Barbora Pafčo, Peter Samaš, Dušan Romportl, Carine Uwamahoro, Jean Bosco Noheri, Adrien Emile Ntwari, Méthode Bahizi, Gaspard Nzayisenga, Julius Nziza, Kirsten Gilardi, Winnie Eckardt, Felix Ndagijimana, Antoine Mudakikwa, Richard Muvunyi, Prosper Uwingeli, Michael Cranfield, Jan Šlapeta, Klára Judita Petrželková, David Modrý

**Affiliations:** 1 Institute of Vertebrate Biology, Czech Academy of Sciences, Brno, Czech Republic; 2 Department of Pathology and Parasitology, Faculty of Veterinary Sciences, University of Veterinary Sciences Brno, Brno, Czech Republic; 3 Department of Physical Geography and Geoecology, Faculty of Science, Charles University, Prague, Czech Republic; 4 Dian Fossey Gorilla Fund, Musanze, Rwanda; 5 Gorilla Doctors (MGVP, Inc.), Davis, CA, USA; 6 Rwanda Development Board, Kigali, Rwanda; 7 Sydney School of Veterinary Science, Faculty of Science, University of Sydney, Sydney, Australia; 8 Institute of Parasitology, Biology Centre, Czech Academy of Sciences, České Budějovice, Czech Republic; 9 Liberec Zoo, Liberec, Czech Republic; 10 Department of Veterinary Sciences, Faculty of Agrobiology, Food and Natural Resources/CINeZ, Czech University of Life Sciences Prague, Prague, Czech Republic; 11 Department of Botany and Zoology, Faculty of Science, Masaryk University, Brno, Czech Republic

**Keywords:** *Anoplocephala*, Anoplocephalidae, *Bertiella*, *Gorilla beringei*, mountain gorilla, parasite ecology, parasite epidemiology, Rwanda

## Abstract

Cestodes of the family Anoplocephalidae parasitize a wide range of usually herbivorous hosts including e.g. rodents, ungulates, primates, elephants and hyraxes. While in some hosts, the epidemiology of the infection is well studied, information is lacking in others. In this study of mountain gorillas in the Virunga Massif, an extensive sample set comprising adult cestodes collected *via* necropsies, proglottids shed in feces, and finally, fecal samples from both night nests and identified individuals were analysed. *Anoplocephala gorillae* was the dominant cestode species detected in night nest samples and individually known gorillas, of which only 1 individual hosted a *Bertiella* sp. It was shown that the 2 species can be distinguished through microscopy based on egg morphology and polymerase chain reaction (PCR) assays for diagnostics of both species were provided. Sequences of mitochondrial (*cox 1*) and nuclear (ITS1, 18S rDNA, 28S rDNA) markers were used to evaluate the phylogenetic position of the 2 cestodes detected in mountain gorillas. Both types of fecal samples, from night nests and from identified individuals, provided comparable information about the prevalence of anoplocephalid cestodes, although the analysis of samples collected from identified gorilla individuals showed significant intra-individual fluctuation of *A. gorillae* egg shedding within a short period. Therefore, multiple samples should be examined to obtain reliable data for wildlife health management programmes, especially when application of anthelmintic treatment is considered. However, while *A. gorillae* is apparently a common symbiont of mountain gorillas, it does not seem to impair the health of its host.

## Graphical abstract

**Figure:**
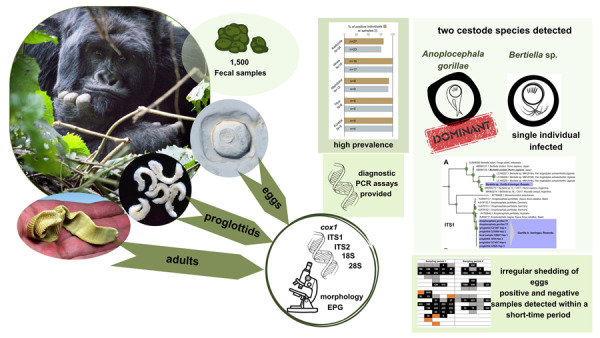

## Introduction

Cestodes of the family Anoplocephalidae which typically use mites as their intermediate hosts parasitize various vertebrates, typically herbivorous, across the globe (Beveridge, 1994; Deplazes *et al*., 2016). The genus *Anoplocephala* Blanchard, 1848 is known from a wide range of hosts including perissodactyls, primates, elephants and hyraxes, which possibly suggests a long association between hosts and parasites (Beveridge, 1994). On the other hand, there is considerable evidence for multiple host switching in the taxa parasitizing rodents and transfer among primates, dermopterans and marsupials has been recorded for the members of the genus *Bertiella* Stiles & Hassall, 1902 and *Progamotaenia* Nybelin, 1917 (Beveridge, 1985*a*, 1994; Haukisalmi *et al*., 2004, 2008, 2009; Wickström *et al*., 2005; Hardman *et al*., 2012). These cestodes are characterized mainly by an absent rostellum and unarmed suckers, a condition that is probably plesiomorphic and the 4 subfamilies could be a polyphyletic assemblage of taxa (Beveridge, 1994) as already confirmed by phylogeny of partial *cox1* (Sharma *et al*., 2016).

Besides a range of studies on anoplocephalids in Arctic rodents (Haukisalmi *et al*., 2001, 2004, 2008, 2009; Wickström *et al*., 2005) and Australian marsupials (Beveridge, 1985*a*, 1985*b*; Beveridge *et al*., 2007; Hardman *et al*., 2012), *Anoplocephala perfoliata* infections have been well studied in horses due to growing incidence of resulting disease (Meana *et al*., 2005; Back *et al*., 2013). Great apes, including humans, and other primates from South America, Africa and South-East Asia are most commonly hosts of *Bertiella*, *Anoplocephala* and *Moniezia* Blanchard, 1891, but occasional infections by *Mathevotaenia* Akhumian, 1946 and *Thysanotaenia* Beddard, 1911 have also been reported (Beveridge, 1994; Sapp and Bradbury, 2020). Occurrence of anoplocephalids in free-ranging non-human primate populations is usually not associated with clinical disease (Doležalová, 2018; Sapp and Bradbury, 2020), although gastrointestinal disturbances, diarrhoea, abdominal pain, anorexia, weight loss, vomit and constipation are reported from humans (Sapp and Bradbury, 2020).

In 1927, Nybelin described *Anoplocephala gorillae* based on material collected from a necropsied mountain gorilla (*Gorilla beringei beringei*) in the region of Mount Sabiniyo in Virunga range (Nybelin, 1927). Ever since, the parasite has been reported in all parasite surveys conducted in both the Bwindi and Virunga mountain gorilla populations, based mainly on presence of typical anoplocephalid eggs and/or detection of proglottids (Redmond, 1983; Ashford *et al*., 1990, 1996; Hastings *et al*., 1992; Sleeman *et al*., 2000; Mudakikwa *et al*., 2001; Kalema-Zikusoka *et al*., 2005; Rothman *et al*., 2008; Petrželková *et al*., 2021). Certain aspects of the ecology/epidemiology of cestode infection were studied in the Bwindi population by Ashford *et al*. (1996). However, only Doležalová *et al*. (2015) attempted to characterize mountain gorilla cestodes using DNA markers supporting identification at the species-level. The sequences of nuclear (ITS2 and 28S rRNA) and mitochondrial (*cox1* and *nad1*) genes obtained from anoplocephalid eggs from mountain gorilla feces clustered with primate *Bertiella* spp., and thus opened a question about spectrum of anoplocephalid cestodes occurring in mountain gorillas (Doležalová *et al*., 2015).

Our overall research has aimed to better understand the degree to which parasite burdens contribute to morbidity and mortality in this endangered great ape and patterns of helminth infections have been analysed across the mountain gorilla population (Petrželková *et al*., 2021, 2022). While strongylid egg counts significantly differed among individual sectors and vegetation types, no clear patterns were found for cestode infections (Petrželková *et al*., 2021, 2022). Therefore, the present study aimed to provide a deeper insight into selected aspects of the tapeworm infections in a sub-population of *G. beringei beringei* in the Volcanoes National Park (VoNP), the Rwandan part of the Virunga Massif. The specific aims of the study were: (i) improvement of diagnostic tools for detection of anoplocephalid cestodes in mountain gorillas, (ii) determining and characterizing the anoplocephalid cestodes in mountain gorillas based on adults collected from necropsies and proglottids and eggs shed in feces, including morphological features and genetic diversity, and, (iii) describing the epidemiology of the infections by analysing the prevalence and variation in egg shedding within and among the mountain gorilla individuals and groups in the VoNP, Rwanda.

## Materials and methods

### Study site

**Figure 1.**
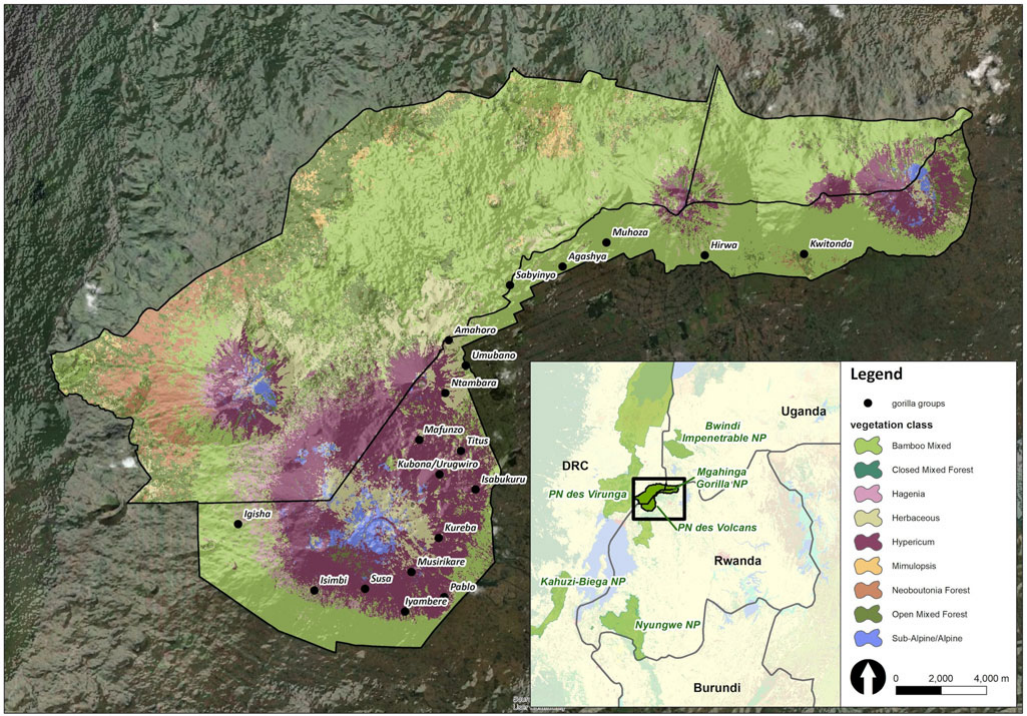
Map of Virunga Massif region picturing the studied gorilla group distribution and vegetation types. Adapted from Petrželková *et al*. (2021).

The study was conducted in the VoNP, the Rwandan section of a complex of protected areas spanning the borders of Rwanda, Uganda and the Democratic Republic of the Congo (DRC) called the Virunga Massif (451 km^2^). The habitat in the VoNP has been characterized in detail elsewhere (Owiunji *et al*., 2005; Hickey *et al*., 2019; Petrželková *et al*., 2021), but briefly, the altitude, ranging between 1600 and 4500 m, determines various vegetation types (Fig. 1) and gorillas normally range in zones where temperatures drop to 0°C. The park's climate consists of 2 rainy (March–May and September–November) and 2 dry seasons (December–February and June–August). The VoNP area is surrounded by a dense human population of up to 1000 people km^−2^ (Bush *et al*., 2010), whereas the mountain gorilla density in the VoNP is estimated to be 1.7 individuals per km^2^ (Hickey *et al*., 2019).

### Fecal sample collection

Nineteen habituated mountain gorilla groups ranging in the VoNP with an estimated 278 individually known gorillas were targeted in 2018–2019. The location, demography, and behaviour of these groups are monitored on a daily basis by the Dian Fossey Gorilla Fund (DFGF) and Rwanda Development Board (RDB) park rangers, while the health of the gorillas is regularly monitored by Gorilla Doctors (GD) veterinarians and daily by park personnel (DFGF and RDB) trained by the GD. GD also provide *in situ* veterinary care to mountain gorillas. Data on group size (number of individuals), age and sex of individually sampled gorillas were obtained from RDB and DFGF.

In 2018 and 2019, fresh fecal samples were collected from habituated mountain gorilla groups by trained staff of RDB, DFGF and GD across the VoNP in 4 sampling periods: in the dry seasons (December–February and June–August), and in the wet seasons (March–May and September–November). Two types of fecal samples were collected: night nest samples from the previous night and samples from identified individuals obtained during group visits. The methodology of nest and individual sample collection is described in detail elsewhere (Petrželková *et al*., 2021; Sinayitutse *et al*., 2021). All samples were placed into plastic bags, labelled with date and time of collection and group name. The samples were also labelled with name of the individual (when available) and sex/age class (dominant silverback/silverback >12 years; blackback 8–12 years; adult >8 years; subadult/juvenile 3.5–8 years; infant 0–3.5 years) (Sinayitutse *et al*., 2021).

**Figure 2.**
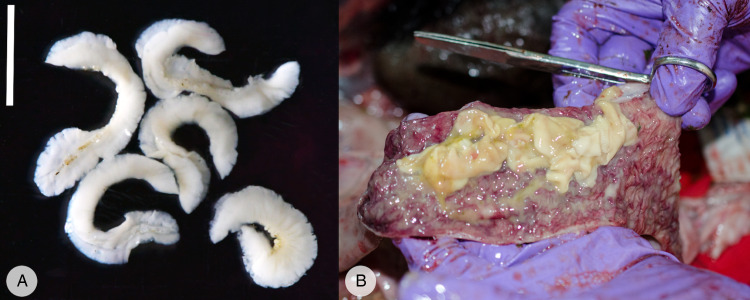
Anoplocephalid cestodes collected from mountain gorillas. (A) Individual proglottids recovered from mountain gorilla feces. Scale 1 cm. (B) Adults inside the mountain gorilla small intestine as recovered at necropsy.

Fecal samples (*n* = 1500) were immediately transported to the GD field laboratory in Musanze, stored in a refrigerator, and examined within a maximum of 48 hours after defecation. Any unusual appearance of the feces, such as diarrhoea or presence of tapeworm proglottids (Fig. 2A) was recorded. Proglottids found in feces were collected and stored in 96% pure ethanol. Three sub-samples of feces were taken: (i) 1 g of feces was preserved in 96% ethanol for DNA analyses (ii) a total of 3 g of feces were preserved in formalin for storage and potential further microscopic examination; and (iii) the last aliquot was immediately processed by Mini FLOTAC^©^ (see below).

### Coproscopic analyses and parasite quantification

Anoplocephalid tapeworm eggs in fresh feces were quantified *in situ* by Mini-FLOTAC^©^ as described in Petrželková *et al*. (2021). The egg counts expressed as eggs per gram of feces (EPG) were recorded. When a sample was evaluated as cestode-negative by Mini-FLOTAC^©^, the formalin-preserved aliquot was re-examined by a sedimentation-based technique (Doležalová *et al*., 2018) providing the quantification as eggs per gram of fecal sediment (EPGS). The EPGS is not equal to the EPG as the fecal sediment obtained after sieving of the feces does not contain the bigger, usually fibrous particles. In our experience, EPGS is about 1.7 times higher than EPG (data not shown). We took the weight of fecal sediment during the Mini-FLOTAC^©^ examination, which enabled us to calculate EPGS also for the samples examined *in situ* by Mini-FLOTAC^©^.

### Egg morphology analyses

**Figure 3.**
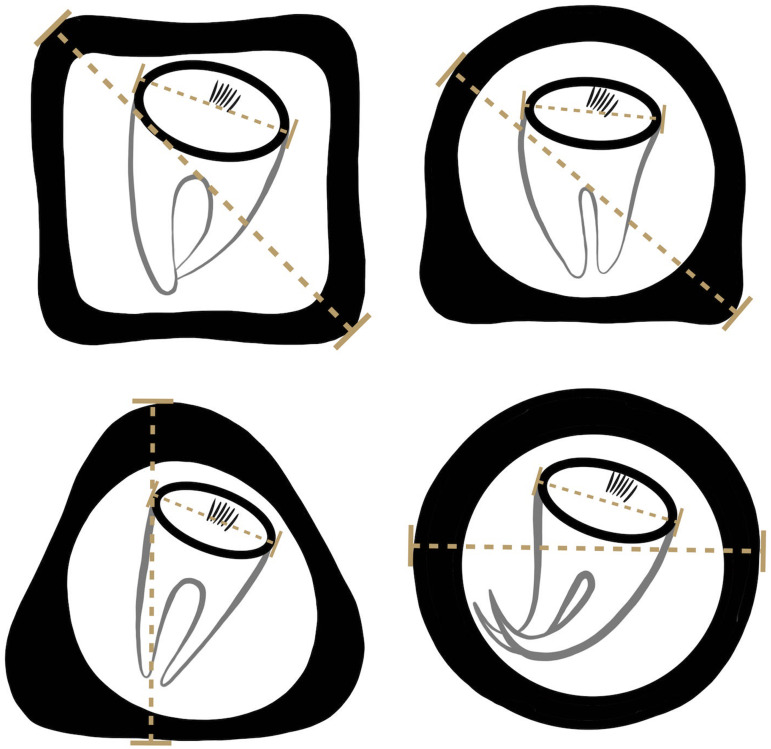
Depicting of how the measurements were taken from anoplocephalid eggs. As many anoplocephalid eggs are asymmetrical, egg and oncospherical diagonals (dashed lines) were introduced as maximal dimensions used for morphology investigation.

Formalin-preserved aliquots of samples identified as *A. gorillae* or *Bertiella* sp. positive based on DNA barcoding (see below) were used for detailed egg morphology investigation. Modified Sheather's sugar flotation technique (Jirků-Pomajbíková and Hůzová, 2018) was used to concentrate the eggs. Egg measurements and microphotographs were taken using an Olympus BX53 microscope equipped with Nomarski differential contrast, an Olympus DP73 digital camera and CellSense Dimension software (Olympus, Tokyo, Japan). Maximum egg and oncosphere dimensions (Fig. 3) and type of pyriform apparatus (PA) classified using a scale from 1 to 10, where 1 refers to *Bertiella*-like PA (brush like, forming thin filaments), whereas 10 refers to the *Anoplocephala*-like PA (bifurcated), were recorded. To evaluate the morphological differences between *Anoplocephala* and *Bertiella* eggs statistically, we used a general linear mixed model with normal distribution (hereafter: LMM).

### Adult cestodes collection

As a part of veterinary surveillance, all deceased animals that are recovered from the forest are necropsied by GD veterinarians following standardized protocols. Various tissue and other biological samples (e.g., ingesta, feces, urine) are collected, including adult parasites observed anywhere in the carcass (Fig. 2B). For purposes of this study, a collection of 53 adult cestodes (obtained from 5 gorillas between 2015 and 2018) from the GD biobank was available. Adult anoplocephalid cestodes were collected mainly from the small intestine, but also from the caecum and colon. The specimens were subsequently preserved in 96% ethanol or 10% formalin. The morphology of adults was studied using a stereomicroscope Olympus SZ51 and a microscope Olympus BX53 (Olympus, Tokyo, Japan). The specimens were determined using taxonomic keys and available publications (Nybelin, 1927; Stunkard, 1962; Beveridge, 1994; Sapp and Bradbury, 2020; Servián *et al*., 2020).

### DNA extraction

DNA was extracted from feces, from proglottids obtained from gorilla feces and from adult cestodes, all stored in ethanol. For fecal samples, aliquots of 0.5 g of feces were put in a thermoblock at 37°C overnight for ethanol evaporation. Then, the DNA was extracted using the DNeasy PowerSoil Pro Kit (Qiagen, Hilden, Germany), with a single modification to the manufacturer's instructions: the incubation steps were prolonged to 30 min. For the proglottids and adult cestodes, approximately 1 cm of proglottid tissue was minced and the total genomic DNA was extracted by NucleoSpin Tissue Kit (Macherey–Nagel, Düren, Germany). The tissue was lysed in Proteinase K overnight and the DNA was eluted to 100 *μ*L of elution buffer. An adult of *A. perfoliata* from a horse from the Czech Republic was used as comparative material. This tapeworm was obtained during a standard necropsy performed at the Department of Pathology and Parasitology, Faculty of Veterinary Medicine, University of Veterinary Sciences Brno and preserved in 96% ethanol until further processing.

### Nuclear ribosomal and mitochondrial DNA amplification

DNA extracted from adult cestodes was used for amplification of partial small ribosomal subunit gene (18S rDNA) and the region spanning the internal transcribed spacer 1 (ITS1), 5.8S rRNA (5.8S) gene, and internal transcribed spacer 2 (ITS2). Partial 18S rDNA and complete ITS1 were amplified in all collected proglottids. A subset of proglottids obtained from feces and adult tapeworms was used for amplification of partial cytochrome c oxidase subunit 1 gene (*cox1*) and gene for large ribosomal subunit (28S rDNA). Detailed information on primers, polymerase chain reaction (PCR) reaction composition and cycling conditions is provided in supplementary files, Table ST1. All PCR products were separated in 1% agarose gel with Midori Green (NIPPON Genetics EUROPE, Germany) and visualized on an UV illuminator. The PCR products were purified using Gel/PCR DNA Fragments Kit (Geneaid, New Taipei City, Taiwan) either directly or from bands cut from the gel and sent for commercial Sanger sequencing (Macrogen Europe B.V., Amsterdam, Netherlands).

### PCR assays for detection of anoplocephalid tapeworms

The nested PCR protocol described by Drogemuller *et al*. (2004) for *A. perfoliata* was tested using the DNA extracted from 2 adult gorilla tapeworms and 2 proglottids from feces as the source of template DNA. The external primers S18 (F) and L3 T (R) amplify the whole ITS1, 5.8S and ITS2 region, while the internal primers AP-ITS2-2F and AP-ITS2-3R target ITS2 only. The PCR reaction composition and cycling conditions are specified in supplementary material (Table ST1). When a band of corresponding size and Sanger sequencing confirmed a successful DNA amplification from adult cestodes, a set of 10 samples of DNA extracted from fecal samples with various anoplocephalid cestode EPG scores (>1000 EPG, *n* = 4; <100 EPG, *n* = 6) was used to test applicability of the assay on fecal DNA. All amplicons were sequenced to confirm the specificity of the assay.

While testing this PCR assay, 1 sample did not produce any band, despite having a high EPG value. Using less specific primers targeting the ITS1 region (McLennan *et al*., 2017) (Table ST1), we obtained a 300 bp amplicon sequence that revealed presence of a *Bertiella* sp. in this sample (see details in the Results section, Chapter ‘*Bertiella* sp. detected in one mountain gorilla individual’). Using newly obtained *Bertiella* and *Anoplocephala* sequences and sequences of *Anoplocephala* and *Bertiella* spp. available in GenBank, *Bertiella*-specific primers were designed in Geneious Prime v.2021.0.1 (https://www.geneious.com). The PCR assay was optimized, and its specificity tested by using the DNA from *Anoplocephala* proglottids and from *Anoplocephala*-positive and *Bertiella*-positive fecal samples (Table ST2).

A subset of cestode-negative samples as revealed by Mini-FLOTAC^©^ examination was examined by both PCR assays and sedimentation method (samples listed in supplementary material Table ST2) to compare the sensitivity of both diagnostic approaches.

### Sequences and phylogenetic analyses

All sequences obtained were checked for quality and trimmed manually using Geneious Prime v.2021.0.1 (https://www.geneious.com). The identity of sequences was validated using the BLAST software (Basic Local Alignment Search Tool; Altschul *et al*., 1990). The alignments were performed by Clustal Omega implemented in Geneious Prime and the same software was used for calculation of pairwise sequence distances. For the phylogenetic analyses, sequences of available anoplocephalid tapeworms (18S rDNA, 28S rDNA, ITS1, *cox1*) were downloaded from GenBank and aligned with our sequences (Supplementary file SF5). The maximum-likelihood phylogenetic trees were calculated by IQ-TREE (Trifinopoulos *et al*., 2016). The most suitable model was chosen by ModelFinder (Kalyaanamoorthy *et al*., 2017) implemented in IQ-TREE based on the highest Bayesian information criterion scores and weights. The tree topology was tested by 1000 replicates of ultrafast bootstrap (Minh *et al*., 2013) and Shimodaira–Hasegawa (SH)-like approximate likelihood ratio test (Anisimova *et al*., 2011). The trees were visualized using the software iTOL v6 (Letunic and Bork, 2021).

## Results

### Proglottids and adult cestodes confirmed to be *A. gorillae*

**Figure 4.**
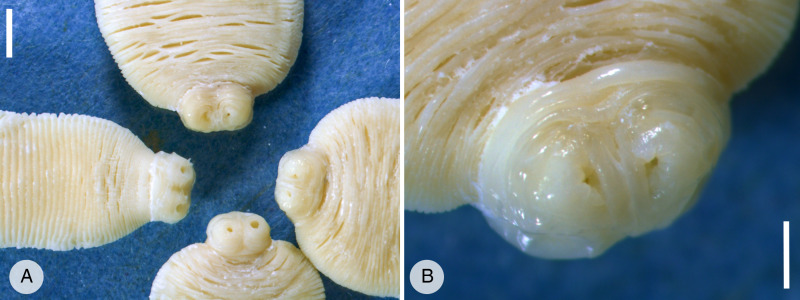
Adult *Anoplocephala* recovered from the mountain gorilla small intestines and preserved in ethanol. (A) Scolexes with 4 circular suckers, very short neck and proximal part of strobila. Note the craspedote proglottids, which are significantly wider than longer. Scale 0.5 cm. (B) Detail of scolex with circular suckers. No rudiments of a rostellum are visible. Scale 2 mm.

Fifty-three adult tapeworms originating from 5 individual gorillas were examined. The length of strobilae varied between 1.5 and 13 cm by 0.8–1.4 cm in width. Four circular suckers (2 mm in diameter) were observed terminally on a small scolex (Fig. 4A). The proglottids in the strobilae were extended transversely, significantly wider than longer (length 0.5–2 mm, width 8–14 mm) and craspedote. No signs of a rostellum were observed (Fig. 4B). The individual proglottids collected from feces were usually curled or folded (Fig. 2A), on average 4 mm long and 15 mm wide, thin and had undulated edges. A collection of specimens used in this study was deposited to the Museum of Environment, Karongi District, Rwanda.

**Figure 5.**
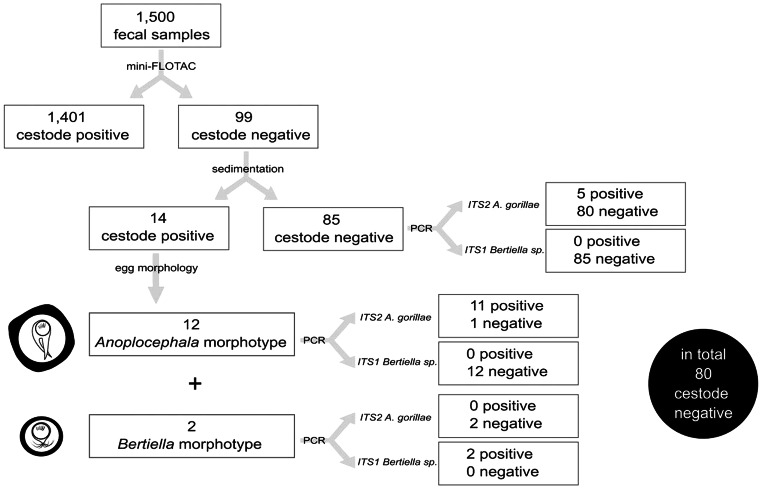
Diagram picturing the workflow of the anoplocephalid cestode diagnostic procedure in mountain gorilla fecal samples. Out of 99 Mini-FLOTAC^©^-cestode-negative samples, 19 were found as falsely-negative.

In total, 1500 fecal samples were collected in 2018 and 2019 (Fig. 5), and 69 proglottids from 69 fecal samples were retrieved. More proglottids in 1 fecal sample were observed sometimes, however the number of proglottids was not recorded and only 1 proglottid per sample was preserved. Five proglottids were compressed between 2 microscope slides to examine the eggs, but no eggs were observed. Single genital pore was distinguished in all 5 proglottids. All listed morphological features corresponded to the morphology of *A. gorillae* as described by Nybelin (1927). Egg counts in the fecal samples containing proglottids varied from 15 to 8190 EPG (eggs per gram of feces) and most feces looked normal (i.e., not diarrhoeic or haemorrhagic), only in 1 case, presence of mucus was recorded.

### PCR detected more cestode-positive samples than microscopy

The nested-PCR assay originally designed to target the ITS2 region of *A. perfoliata* was efficient in *A. gorillae* DNA amplification, despite 1 mismatch in each of the internal primers. Amplicons of expected size were obtained using DNA extracted from samples with 8 to 4260 EPG and no non-specific amplification was detected. When using PCR assay targeting *Bertiella* ITS1 region, amplicons of expected size were obtained from samples containing *Bertiella* sp. eggs, while no amplicon was observed using DNA extracted from *Anoplocephala* spp. proglottids or from fecal samples containing *A. gorillae* DNA. Protocols for both PCR assays are specified in supplementary material Table S1.

Out of the 99 cestode-negative samples as detected by Mini-FLOTAC^©^, the sedimentation method revealed 14 samples containing cestode eggs in quantities ranging from 5 to 2139 eggs per gram of fecal sediment (EPGS) (Table S2, Fig. 5). In 12 of the 14 samples, egg morphology corresponded to *A. gorillae*, while eggs in the remaining 2 samples were of the *Bertiella* morphotype (see chapter on *Anoplocephala* and *Bertiella* egg morphology).

PCR assay targeting *Anoplocephala* detected 16 positive samples, of which 5 samples were initially evaluated as cestode negative by sedimentation (i.e., negative by 2 microscopy-based methods), while in the other 11 PCR-positive samples, *Anoplocephala* eggs were observed using the same technique (Fig. 5).

All amplicons obtained from fecal DNA samples were sequenced and were identical with ITS2 sequence obtained from adult *A. gorillae*. In 1 sample evaluated as *Anoplocephala*-positive by sedimentation (5 EPGS), no amplicon was obtained despite an increased effort (DNA was re-extracted, PCR was repeated with both increased and decreased DNA template volume).

Using the PCR assay targeting *Bertiella* ITS1, only 2 samples were detected as *Bertiella*-positive and in both, *Bertiella-*morphotype eggs were observed also in the sedimentation (Fig. 5). Sequences obtained from these samples were identical with the *Bertiella* sp. sequences from the original fecal sample (see the next chapter). In all remaining 97 samples, *Bertiella* DNA was not detected.

To conclude, in total 19 falsely cestode negative samples were detected, 14 *via* sedimentation method, while PCR (both assays combined) identified 18 false negatives, including 5 samples evaluated as negative in sedimentation. The information about the 19 false negatives was used to complement the anoplocephalid cestode prevalence calculations.

### *Bertiella* sp. detected in 1 mountain gorilla individual

While initially optimizing PCR assays for the detection of *A. gorillae*, no amplicon was obtained by *Anoplocephala*-specific primers in 1 sample, despite the high egg counts recorded. Primers targeting the ITS1 region of anoplocephalid tapeworms (McLennan *et al*., 2017) amplified a sequence (683 bp) which differed by 41.1% from ITS1 sequences obtained from our *A. gorillae* proglottids and closely matched *Bertiella* sp. H_1 (Accession number MK993273.1, host: human, country: Argentina, 90.96% identity) in BLAST. Additionally, partial 18S rDNA (714 bp) was also amplified and sequenced from the sample to allow a better characterization of this taxon. In this nuclear marker, the pairwise sequence distance between the newly detected *Bertiella* sp. and *A. gorillae* was 18.5%. The formalin-preserved aliquot of this sample was re-examined by flotation, showing eggs with morphological features corresponding to *Bertiella* sp. as described below.

The *Bertiella*-positive sample originated from a mountain gorilla male infant named Inkingi from the Hirwa group. Firstly, all samples collected from this individual (*n* = 4; collected in 2 sampling periods 3 months apart) were tested to verify that the *Bertiella* eggs in feces were not just a spurious parasite. *Anoplocephala*-specific PCR assay was also applied to detect possible co-infection by both cestode species. Two Inkingi's samples were positive for *Bertiella* only, but both *A. gorillae* and *Bertiella* sp. DNA was detected in the remaining 2 samples. Subsequently, fecal samples from the other 15 members of Inkingi's family group, were tested for presence of *A. gorillae* and *Bertiella* sp. by PCR, using 2 samples with the highest EPG from each gorilla. All individuals tested positive for *A. gorillae* only. Therefore, *Bertiella* and *A. gorillae* prevalence among the Hirwa group individuals was 6.6 and 100% respectively. Inkingi was the only individual infected by both anoplocephalids. Finally, another 100 samples collected from different individuals from 16 study groups were randomly selected and tested with the *Bertiella*-specific PCR assay; all with negative results.

### ITS1 and 18s rDNA phylogenies confirm 2 anoplocephalid cestodes parasitizing mountain gorillas

Representative sequences of complete ITS1, 5.8S and ITS2 (1876 bp) and partial 18S rDNA (654 bp) were obtained from 2 selected *A. gorillae* adults originating from 2 deceased gorillas from 2 different groups. No difference in either marker was observed. 18S rDNA and ITS1 sequences were also obtained from 51 proglottids. At least one of the markers was sequenced in 14 proglottids (ITS1 and 18S rDNA each in 7 samples), while amplification was not successful in 4 samples even though the DNA was re-extracted, DNA template volume in the PCR was increased and annealing temperature adjusted.

All sequences of 18S rDNA (583–715 bp) were identical to each other except for 1 sequence that differed in a single nucleotide, which was ambiguous (S in position 291 in the alignment). The majority of ITS1 sequences (326–642 bp; *n* = 46) was also identical to the sequence obtained from adults and this haplotype was labelled as Hap1. Five more variants of ITS1 were identified in our dataset (Hap2 – Hap6), each represented by a single sequence. The haplotypes differed in 1 to 6 nucleotides from each other. Eight sequences were too short to be assigned to either haplotype Hap1 or Hap2. ITS1 sequences obtained from fecal samples (*n* = 5) during the optimization of PCR assays were also identical with the haplotype Hap1. The sequences were uploaded to GenBank under accession numbers OR863079-91 and OR863669-74.

**Figure 6.**
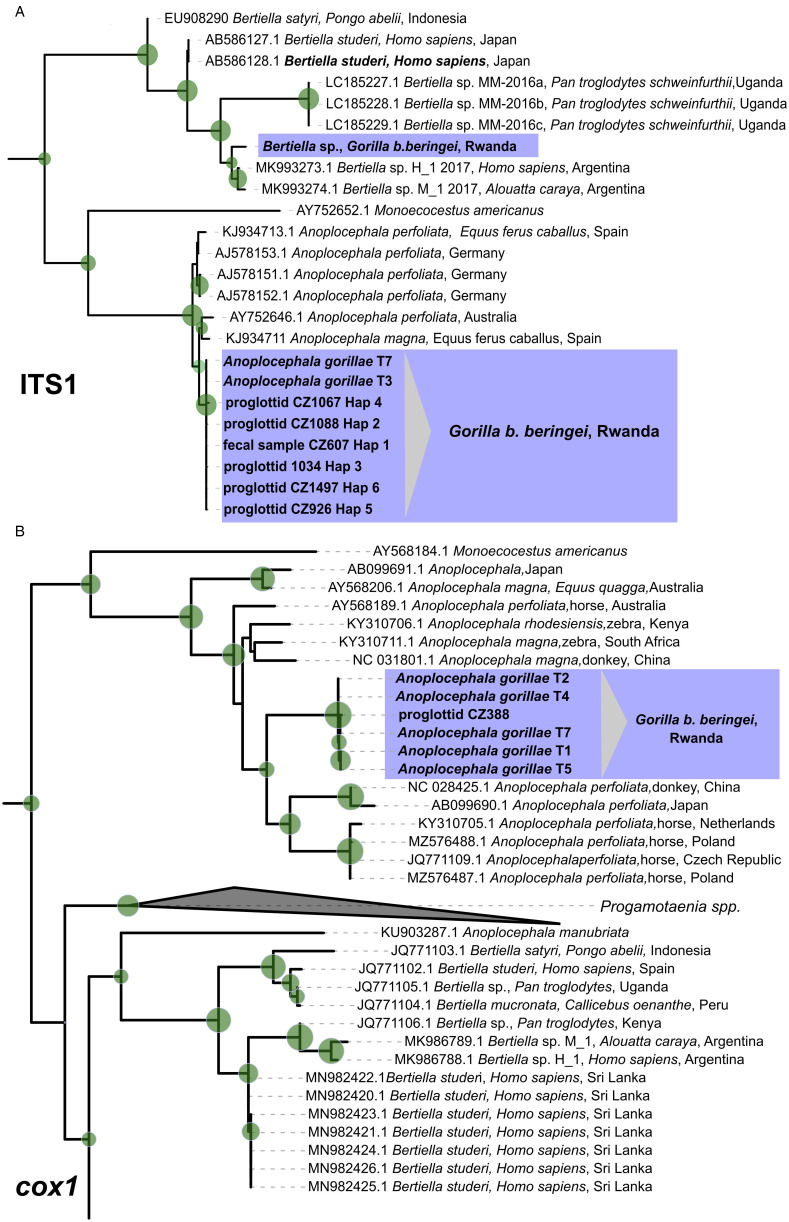
Cut outs of (A) Maximum likelihood phylogenetic tree derived by TPM2 + F + I + G4 model from available ITS1 sequences of anoplocephalid cestodes. Sequence of *Hymenolepis microstoma* (Accession number AJ287525) was used as an outgroup (not shown). (B) Maximum likelihood phylogenetic tree derived by TIM2 + F + R5 model from available *cox1* sequences (1127 bp) of anoplocephalid cestodes. The green circles mark nodes with ultrafast bootstrap and SH-like approximate likelihood ratio test higher than 75%. Size of the circle correlates to the value. Complete uncollapsed trees can be found in Supplementary files SF2 (ITS1) and SF3 (*cox1*). The trees were visualized and edited in iTOL v6 (Letunic and Bork, 2021). Sequences derived in this study are in blue boxes, sequences from GenBank are identified by accession number.

Final alignment of 18S rDNA (897 bp) comprised 6 sequences obtained from *Bertiella* sp. and *A. gorillae* specimens in this study and 22 anoplocephalid cestode sequences available in GenBank. The alignment of ITS1 (1137 bp) consisted of 9 sequences from our *Bertiella* sp. and *A. gorillae* samples and 90 more sequences of anoplocephalid cestodes downloaded from GenBank. The maximum likelihood phylogenetic trees were calculated by TPM2 + F + G4 and TPM2 + F + I + G4 for 18S rDNA and ITS1 respectively (Fig. 6A; Supplementary files SF1, SF2).

In both phylogenetic analyses, distinct clades of *Anoplocephala* spp. and *Bertiella* spp. were formed (Fig. 6A, Supplementary files SF1, SF2). All sequences originating from proglottids, and adult worms clustered with *Anoplocephala* spp. from equids. The difference between *A. gorillae* and *A. perfoliata* was 7.6–13.2 and 2.6–3.5% for ITS1 and 18S rDNA respectively. In the ITS1, *A. gorillae* differed from *A. magna* by 6.3–8.2%. Sequence obtained from the fecal sample with *Bertiella* eggs clustered within a clade comprising other *Bertiella* spp. sequences from non-human primates and humans (Fig. 6A), specifically in a sub-clade with sequences originating from a human and a howler monkey from Argentina, but the difference was about 11.4–11.7%. The sequences from Ugandan chimpanzees (Accession numbers LC185227-9; geographic proximity to our material) differed from our *Bertiella* sp. by 31.2–37.4%. In 18S rDNA phylogenetic tree (Supplementary file SF1), the mountain gorilla *Bertiella* clustered most closely with a sequence from a sanctuary chimpanzee from Kenya (MW741843; Supplementary file SF1), but the lowest pairwise distance (0.4%) was observed between our *Bertiella* and sequence of *B. studeri* from a monkey (most probably a long-tailed macaque) from Mauritius (GU323706). Previously published sequence of *Bertiella* sp. obtained from a mountain gorilla from Rwanda (MW741845) differed from our isolate by 2.1% and clustered in other *Bertiella* sub-clade (Supplementary file SF1).

### Cox1 and 28s rDNA phylogenies confirm position of *A. gorillae* in the genus Anoplocephala

We obtained 11 sequences of partial *cox1* (459–1064 bp) from 9 adult cestodes and 2 proglottids isolated from feces. Four haplotypes differing by 1 to 4 nucleotides were detected. The BLAST search showed 89% similarity to *A. perfoliata* (accession number KR054960) for isolate T7, while the other isolates showed 89.74–89.85% similarity to *A. magna* (accession number KU236385). The sequences were uploaded to GenBank under accession numbers OR864671-76. The maximum likelihood phylogenetic tree (Fig. 6B, Supplementary file SF3) was calculated from an alignment (1127 bp) of 152 anoplocephalid cestode sequences, including 6 sequences representing our isolates from mountain gorilla by TIM2 + F + R5 model with 2 *Hymenolepis* spp. sequences used as outgroup.

**Figure 7.**
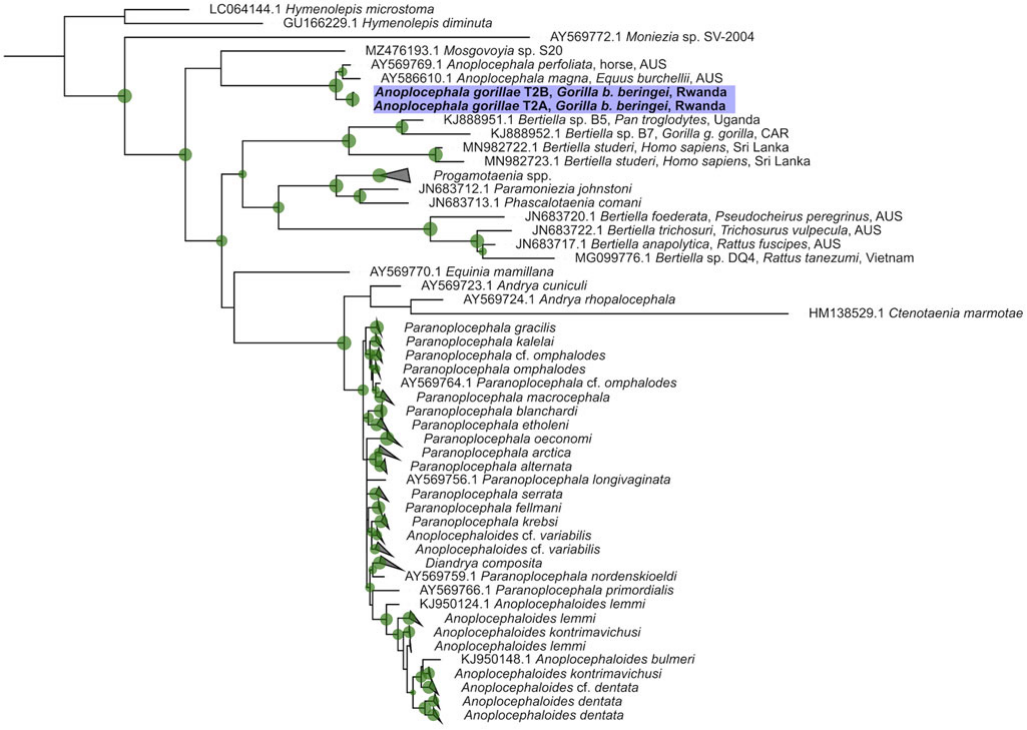
Maximum likelihood phylogenetic tree derived by TPM3u + F + R3 model from 1910 bp alignment of available 28S rDNA anoplocephalid cestode sequences. The green circles mark nodes with ultrafast bootstrap and SH-like approximate likelihood ratio test higher than 75%. Size of the circle correlates to the value. When appropriate, the clades have been collapsed (the uncollapsed tree can be found in Supplementary file SF4). The tree was visualized and edited in iTOL v6 (Letunic and Bork, 2021). Sequences derived in this study are in blue boxes, sequences from GenBank are identified by accession number. AUS, Australia; CAR, Central African Republic.

In total, 5 sequences (1169–2011 bp) of 28S rDNA 5′-end were obtained from 3 adult tapeworms and 2 proglottids collected from feces. All sequences were identical. Only in 1 adult (isolate T2), 2 haplotypes differing in a single nucleotide deletion (G at position 704) were detected. BLAST search showed 97.98–98.05% similarity (71% coverage) to *A. perfoliata* isolate N54 (accession number AY569769). The sequences were uploaded to GenBank under accession numbers OR863675-80. The maximum likelihood phylogenetic tree (Fig. 7, Supplementary file SF4) was calculated from a 1910 bp alignment of 127 anoplocephalid cestode sequences and 2 *Hymenolepis* spp. sequences used as outgroups by the TPM3u + F + R3 model.

In both trees (Fig. 6B, Fig. 7, Supplementary files SF3, SF4), clades corresponding to distinct species were formed. Sequences from mountain gorillas' *A. gorillae* clustered in a separate clade close to other *Anoplocephala* spp. Surprisingly, *cox1* sequence of *A. manubriata* (accession number KU903287) from an Asian elephant did not cluster with other *Anoplocephala* spp. but with *Bertiella* spp. sequences obtained from primates, including humans (Fig. 6B, Supplementary file SF3). In 28S rDNA, the genus *Bertiella* appeared as a polyphyletic group (Fig. 7, Supplementary file SF4) with a clade comprising sequences from primates, including humans and a separate clade comprising sequences from marsupials.

### *Anoplocephala gorillae* and *Bertiella* sp. egg morphology differences summarized

**Figure 8.**
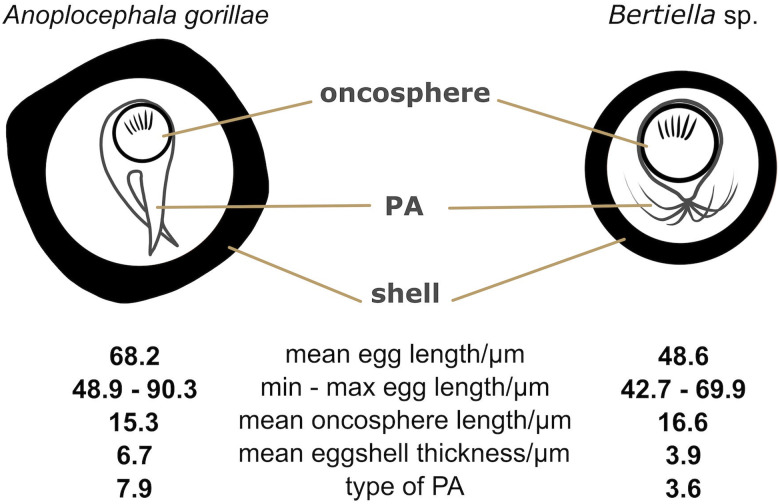
Comparison of *A. gorillae* and *Bertiella* sp. egg morphology. The type of pyriform apparatus (PA) was classified using a scale from 1 to 10, where 1 refers to clearly *Bertiella*-like PA (brush-like), whereas 10 refers to the *Anoplocephala*-like PA (bifurcated).

Three different situations were recorded within our sample set after microscopy and PCR: either (i) *A. gorillae* or (ii) *Bertiella* sp. were found as a mono-infection or (iii) co-infection of both species was detected in a sample. Five samples covering each situation were used for detailed egg morphology examination to investigate if the eggs of the 2 cestodes can be distinguished. In total 261 eggs from 5 gorilla individuals were examined, including 131 *Bertiella* eggs originating from a single gorilla individual and 130 *A. gorillae* eggs extracted from 4 individuals. Based on egg shape, maximum length, oncosphere length and PA, 2 egg morphotypes corresponding to *A. gorillae* and *Bertiella* sp. were distinguished (Fig. 8). *A. gorillae* egg length (mean = 67.0 *μ*m, 95% CI 65.1–69.0, *n* = 130) was larger than in *Bertiella* sp. (mean = 48.2 *μ*m, 95% CI 46.1–50.5; LMM: estimate ± s.e. = −0.020 ± 0.001, *t* = −21.4, *P* = 0.001). Oncosphere length did not differ between *A. gorillae* (mean = 16.0 *μ*m, 95% CI 13.8–19.1) and *Bertiella* sp. (mean = 18.4 *μ*m, 95% CI 14.6–24.8, *n* = 131; LMM: *t* = −1.27, *P* = 0.27). Eggshell thickness had a non-significant tendency to be larger in *A. gorillae* (mean = 6.5 *μ*m, 95% CI 4.8–8.5, *n* = 100) than in *Bertiella* sp. (mean = 3.9 *μ*m, 95% CI 2.0–7.2, *n* = 100; LMM: *t* = −0.63, *P* = 0.08). Similarly, the PA, classified on a scale from 1 (brush-like) to 10 (bifurcated), had a non-significant tendency to be more bifurcated in *A. gorillae* (mean = 8.0, 95% CI 6.7–9.2, *n* = 98) and rather brush-like in *Bertiella* sp. (mean = 3.6, 95% CI − 5.3–12.4, *n* = 99; LMM: *t* = −8.70, *P* = 0.06). Figure 8 summarizes the basic morphological characteristics and morphometry of *Anoplocephala* and *Bertiella* eggs.

**Figure 9.**
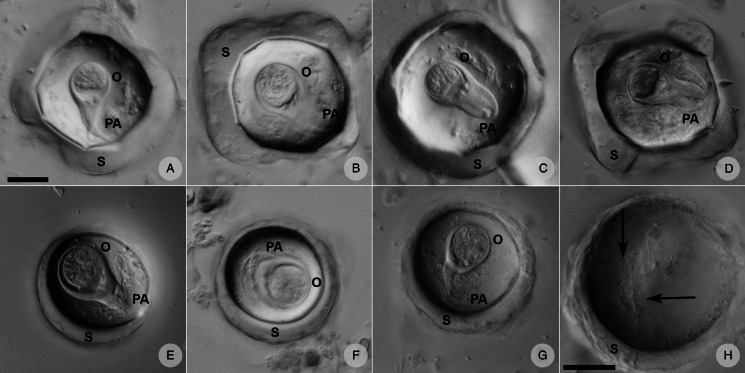
Anoplocephalid cestode eggs observed in mountain gorilla feces. (A—D) *Anoplocephala* morphotype typical for rectangular or polygonal shape, very thick shell (S), oncosphere (O) relatively smaller, and bifurcated pyriform apparatus (PA). (E—G) *Bertiella* morphotype characterized by round shape, thick shell, relatively bigger oncosphere and brush-like PA. (H) Detail of the PA fibres (arrows) visible when focusing throughout the depth of the egg. (A–G) on the same scale, both scales correspond to 20 *μ*m.

Eggs of *A. gorillae* had various shapes, mostly quadrangular with a very thick eggshell, but a triangular or polygonal shape was also recorded. The oncosphere was mostly kidney-shaped surrounded by a clearly bifurcated PA. In *Bertiella* sp., oncospheres were mostly spherical and the egg wall was thinner than observed in eggs of *A*. *gorillae*. The PA of *Bertiella* sp. was branched into many thin fibres, resembling a brush (Fig. 9).

### *Anoplocephala gorillae* eggs detected in all studied groups with very variable EPG

**Figure 10.**
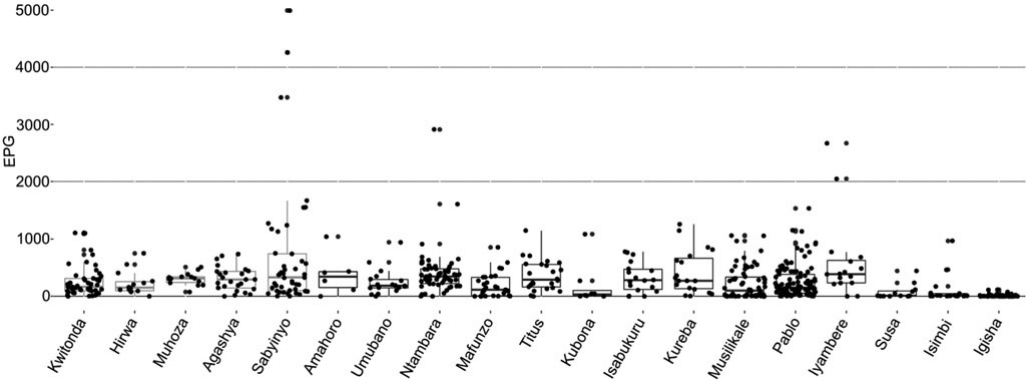
*Anoplocephala gorillae* egg counts in studied mountain gorilla groups expressed as eggs per gram of feces (EPG) including all samples collected between 2018 and 2019. *X* axis shows individual gorilla groups. For statistical analyses of EPG see Petrželková *et al*., 2021.

*Anoplocephala gorillae* eggs were detected in the vast majority (95%) of the 1500 fecal samples examined and the egg counts varied from 8 to 8190 eggs per gram of feces (EPG) with a median of 203 EPG. In total, 5.5% (*N* = 82) of samples exceeded 1000 EPG, and 1.3% (*N* = 20) samples had EPG higher than 2000. We observed a great variability of EPG values within and among groups (Fig. 10). The differences between groups were statistically significant – details in Petrželková *et al*., 2021.

In total, 617 nest samples from 19 groups were collected and examined in 7 sampling events corresponding to dry and wet seasons of 2018 and 2019. However, the majority of groups was sampled only during the dry (Jan–Feb) and wet (Sep–Nov) season in 2018 (382 fecal samples; Table 1). As these samples do not originate from identified individuals, we avoid interpreting the data as prevalence. Considering the results from coproscopy complemented by the PCR results, *A. gorillae* was detected in all 19 groups with percentage of positive samples ranging from 28.5% to 100% and reaching over 70% of positive samples in 15 out of 19 groups (Table 1).

**Table 1.** Percentage of *Anoplocephala gorillae*-positive nest samples (PAPS) in dry (Jan–Feb) and wet (Sep–Oct) seasons by groups in 2018

Group	Group size	Dry		Wet	
		No. of samples	PAPS (%)	No. of samples	PAPS (%)
Kwitonda	30	26	100	23	91.3
Hirwa	17	14	100	17	76.5
Muhoza	8	8	100	6	100
Agashya	14	14	100	11	90.9
Sabyinyo	17	10	100	15	100
Amahoro	19	NA	NA	6	100
Umubano	12	9	100	9	88.9
Ntambara	12	10	100	9	100
Mafunzo	14	NA	NA	11	81.8
Titus	6	6	100	5	100
Kubona	NA	NA	NA	5	80
Isabukuru	13	9	100	9	66.7
Kureba	8	7	100	4	100
Musilikale	18	11	100	12	66.7
Pablo	23	16	93.7	17	94.1
Iyambere	6	4	100	4	100
Susa	18	12	100	16	75
Isimbi	18	9	77.8	8	62.5
Igisha	26	16	62.5	14	28.5

Coproscopy results were corrected by PCR results (adding falsely negative samples). NA – no samples collected.

During 2018–2019, in total 651 fecal samples were collected from 124 identified individuals belonging to 9 gorilla groups. One to eight samples per individual were obtained within 2 sampling periods (4 to 6 weeks). *Anoplocephala gorillae* prevalence in the groups ranged from 76.19 to 100%. The egg counts varied from 8 to 8190 EPG, with an average of 384 EPG and a median of 248 EPG. The youngest *A. gorilla*-positive individual was a 3-month-old infant from Hirwa group (8 EPG). The probability of detecting *A. gorillae*-positive individuals significantly increased with the number of samples examined (logistic regression; estimate = 1.49 ± 0.60, *F*1124 = 36.8, *P* < 0.001). The probability to detect a positive individual increased from 68% to 98% just by increasing the number of samples from 1 to 3. Most individuals (78.6%) were found *A. gorillae*-positive after examination of the first sample, but up to 4 samples were required to detect *A. gorillae* presence.

**Figure 11.**
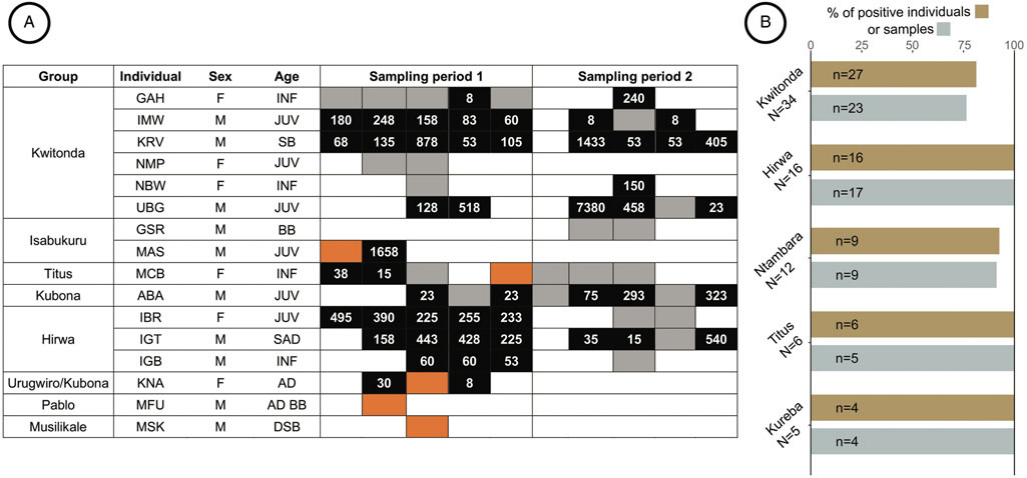
(A) Examples of *Anoplocephala gorillae* EPG fluctuation in selected individuals resampled during 2 sampling periods (May-July 2018 and September-November 2018). Black = *A. gorillae* positive sample with EPG value; grey = *A. gorillae* negative sample; white = animal not sampled; orange = microscopy negative, but PCR positive; M, male; F, female; INF, infant; JUV, juvenile; SAD, subadult; AD, adult; BB, blackback; SB, silverback; DSB, dominant silverback. (B) Comparison of information gained through individual sampling, providing individual prevalence (percentage of positive individuals) and nest sampling, providing percentage of positive samples (PPS). N = total number of individuals in the group, n = number of samples (nest sampling) or individuals examined

In most animals, a considerable fluctuation of EPG values over time was observed in samples collected from the same individual (Fig. 11A). In 9 gorillas, both positive and negative samples occurred within 1 sampling period. In 2 individuals, only 1 sample was collected and found negative by coproscopy, but the PCR showed presence of *A. gorillae* DNA (Fig. 11A).

In 35 samples (5.4%) originating from 22 individuals (17.6%), the EPG exceeded 1000 EPG. These outlying individuals comprised 10 males, 12 females, 13 adults, 2 subadults, 5 juveniles and 2 infants. In most of these animals, only a single sample had such high *A. gorillae* EPG, while in 8 individuals (3 adult males, 2 adult females and 3 juveniles/infants), high *A. gorillae* EPG values were recorded repeatedly. However, repeatability of high EPGs for all individuals sampled more than once was generally low [*r* (95% CI) = 0.22 (0.14–0.30)].

Out of 124 individually sampled gorillas, 17 individuals from 10 different groups and between the age from 15 months and 20 years were *A. gorillae* negative. For the majority of these animals (*N* = 13), only 1 fecal sample was collected and examined. In 3 individuals (1 adult female and 2 infants), 2 samples were collected and examined. Finally, in 1 male juvenile all 3 samples were tested negative.

In 5 groups, sample sets from both, nest and individual sampling, were available from the same period allowing us to compare fecal prevalence (percentage of positive samples) and individual prevalence (percentage of positive individuals). In 3 groups, both prevalence values reached 100%, while in 2 groups, the individual prevalence was slightly higher than fecal prevalence (Fig. 11B).

## Discussion

Using an extensive set of fecal samples, dozens of proglottids, and adult helminths, anoplocephalid cestodes parasitizing mountain gorillas in Rwanda were identified as *A. gorillae* and *Bertiella* sp. and characterized both morphologically and genetically. Specific PCR assays were provided for detection of both the dominant *A. gorillae* and the rarely occurring *Bertiella* sp. Prevalence and individual egg excretion trends of *A. gorillae* were described using fecal samples from identified individuals and nests.

Anoplocephalid cestode species are defined mainly by the morphology of proglottids and the position of genital organs (Beveridge, 1994). Although we did not examine histological sections of mature proglottids, all examined adults exhibited morphological features described by Nybelin (1927) as typical of *A. gorillae*, particularly the suckers, which are terminally attached to a small scolex without any rostellum. Other *Anoplocephala* species have a similar gross morphology, however the DNA sequence analysis showed clear differentiation of *Anoplocephala* from mountain gorillas and *A. perfoliata*, *A. magna, A. rhodesiensis* from equids and *A. manubriata* from elephants. Since *A. gorillae* is the only species of the genus described from mountain gorillas, we concluded that the cestodes found in mountain gorilla necropsies are *A. gorillae*, as named by Nybelin (1927). The 18S rDNA and ITS1 sequences obtained from all proglottids shed in feces matched the reference sequences obtained from the identified adult cestodes, and in the vast majority of the fecal samples examined, we detected *A. gorillae* DNA. However, we also found 1 gorilla infant that harboured *A. gorillae* along with an undetermined *Bertiella* sp.

*Bertiella* is another anoplocephalid cestode genus that commonly parasitizes primates, including humans, but also rodents, marsupials and dermopterans (Beveridge, 1994; Wickström *et al*., 2005; Beveridge *et al*., 2007; Hardman *et al*., 2012; Sapp and Bradbury, 2020) and apparently also mountain gorillas, although rarely. The only material available for the identification of this *Bertiella* found in a single mountain gorilla individual was the eggs. Anoplocephalid cestode eggs are typical for the presence of a PA, which encloses the oncosphere (Beveridge, 1994), and egg morphology can be used to distinguish many of the genera within the family (e.g. Conn, 1985; Beveridge, 1985*b*; Galán-Puchades *et al*., 2000; Haukisalmi and Henttonen, 2000; Tenora *et al*., 2002), sometimes even species (Bohórquez *et al*., 2014). Detailed morphometric analysis of the 2 anoplocephalid cestode species detected in the Virunga mountain gorillas revealed that *A. gorillae* can be reliably distinguished from *Bertiella* sp. by a polymorphic shape ranging from triangular to polygonal, a larger absolute size reaching up to 90 *μ*m, proportionally smaller oncosphere, and PA clearly bifurcated into only 2 branches. In contrast, *Bertiella* eggs are typically spherical or subspherical. Due to the smaller size of the egg, the oncosphere occupies a larger area of the egg and eventually, the PA branches into numerous fine, wavy filaments. It should be noted that the filamentous character of PA is typical for *Bertiella* spp. in primates, while in other hosts, such as marsupials, both filamentous as well as paired PA occur commonly (Beveridge, 1985*a*). The presence of *Bertiella* in our sample set would not have been noticed without discrepancies in PCR results, demonstrating the need for personnel conducting the microscopy to be properly trained and experienced. In an ideal case, simultaneous DNA-based diagnostic techniques should be performed.

Although a number of *Bertiella* spp. sequences are available in GenBank, none of them showed conspecificity with our mountain gorilla isolate, and we are not able to assign our *Bertiella* sp. isolate to any sequenced species. Surprisingly, analyses of the nuclear ITS1 sequence showed that our *Bertiella* from Virunga gorillas is genetically closer to the South American isolates (about 11% difference) than to the Ugandan isolates (more than 30% difference), which is in agreement with the results of Doležalová *et al*. (2015) who showed a close relationship between isolates from Peru, Brazil and Kenya with an overall genetic distance of 0.2–12% based on *cox1*. Unfortunately, we were not able to obtain *cox1* sequences from our sample and could not compare the results with the latter study.

While 29 species of *Bertiella* have been described (Beveridge, 1994), sequences from only 3 primate-associated species (*B. mucronata*, *B. satyri*, *B. studeri*) and 4 other species from rodents and marsupials (*B. affinis*, *B. anapolytica*, *B. foederata* and *B. trichosuri*) are available in GenBank. More than half of the *Bertiella* spp. sequences available in database belong to undetermined species, mostly from primates. Such a lack of sequences may impact the topology of phylogenetic trees in our ITS1 analysis. While multiple markers have been sequenced for *Bertiella* spp. from primates, only 28S rDNA sequences of *Bertiella* spp. from rodents and marsupials have been obtained. The sequences of additional markers are necessary to confirm the polyphyly of the genus *Bertiella* as seen in the 28S rDNA phylogenetic tree, where the sequences from primate parasites form clades separated from *Bertiella* spp. parasitizing rodents and marsupials by clades of *Progamotaenia* spp., *Paramoniezia johnstoni* and *Phascalotaenia comani*. This polyphyly would support separation of the *Bertiella* spp. parasitizing in primates from species occurring in marsupials, rodents and dermopterans as suggested by Baer (1927) who suggested the genus *Prototaenia* for the latter group, based mainly on the level of cirrus sac development and fine differences of other reproductive organs. These distinctions were subsequently deemed as insufficient for classification of a separate genus by Baer (1934) and thus, single genus *Bertiella* has been accepted ever since (Beveridge, 1994). Additional sequences from properly determined *Bertiella* species will help clarify the phylogeography and taxonomy of the genus.

The study by Doležalová *et al*. (2015) also published a sequence of *Anoplocephala* cf. *gorillae* (isolated from eggs in a single fecal sample), but the sequence clustered within the *Bertiella* clade. The authors suggested that *A. gorillae* should be moved to the genus *Bertiella*. In their study, sequences of 5.8S-ITS2 region (JQ771100), *nad1* (JQ771117) and 18S rDNA (MW741845) were obtained from this particular cestode referred to as *A. gorillae* and both ITS2 region and 18S rDNA differed significantly from our *A. gorillae* sequences (60.3% and 18% respectively). Moreover, our *Bertiella* 18S rDNA sequence clustered in a different subclade than the cestode from a mountain gorilla sequenced by Doležalová *et al*. (2015) (Supplementary file SF1) and the pairwise sequence distance between the 2 sequences was 2.1%. Considering that our results are based on a solid sample set and that the previous study used only 1 fecal sample as DNA source, we can conclude that *A. gorillae* indeed is a member of *Anoplocephala* and that it was presumably another *Bertiella* sp. that was sequenced in the previous study. As the *Bertiella* spp. seemed to be rather rare, the mountain gorillas are most probably infected by an occasional spill-over from different hosts, as observed in *Bertiella* and other species within the Anoplocephalinae (Beveridge, 1994; Haukisalmi *et al*., 2004, 2008, 2009; Wickström *et al*., 2005; Hardman *et al*., 2012).

Microscopy following flotation or sedimentation techniques represents the most common approach to routine coproscopic detection of metazoan gastrointestinal parasites in wildlife (Masi *et al*., 2012; Vimalraj and Jayathangaraj, 2015; Naz *et al*., 2021; Petrželková *et al*., 2021) due to its cost effectiveness and simplicity. However, sensitivity of these methods and challenging identification of detected parasites commonly lead to biased results. In our sample set, all fecal samples scored as negative using Mini-FLOTAC^©^ were re-examined by sedimentation and PCR, which combined detected almost 20% of these samples containing either *A. gorillae* eggs or DNA. Egg numbers determined *via* sedimentation in these falsely negative samples ranged from very low (5 EPGS) to very high, reaching over 2000 EPGS. Such discrepancy may be caused partially by slightly higher sensitivity of the sedimentation method, which was 5 EPGS in our case, while the threshold for Mini-FLOTAC^©^ was 8 EPG. On the other hand, it is not surprising that PCR detected falsely negative samples from microscopy, as eggs might be shed irregularly, while the parasite DNA is usually present in the amount sufficient for molecular detection (Wong *et al*., 2014; Rijsman *et al*., 2016) Nevertheless, in 1 case, we were not able to amplify *A. gorillae* DNA using PCR despite the eggs observed in sedimentation, though in low numbers (5 EPGS). The falsely negative samples represented only 1.3% of the total sample set and 1.5% of samples from identified individuals and, therefore, their effect on overall results is marginal. However, 2 false-negative samples were the only samples collected from 2 gorilla individuals and therefore, without PCR, these 2 gorilla males would be considered as *A. gorillae* negative.

Even though there are apparently 2 anoplocephalid cestodes parasitizing Rwandan mountain gorillas, *Bertiella* sp. was detected in a single individual, moreover in co-infection with *A. gorillae*. Thus, the epidemiological and ecological data of this mountain gorilla subpopulation can be related to *A. gorillae*. This cestode is a common symbiont in the intestine of mountain gorillas a few months after birth. Previous studies also reported a high percentage of cestode-positive fecal samples in both Virunga and Bwindi populations (Redmond, 1983; Ashford *et al*., 1996; Sleeman *et al*., 2000; Kalema-Zikusoka *et al*., 2005). This situation contrasts strongly with other African great apes where cestode infections are only rarely detected (Ashford *et al*., 2000; Lilly *et al*., 2002; Krief *et al*., 2005; Kooriyama *et al*., 2012; McLennan and Huffman, 2012; Sá *et al*., 2013; Drakulovski *et al*., 2014; Pafčo *et al*., 2017; Vlčková *et al*., 2018) even though seasonal peaks of infections were observed (Wrangham, 1995). Occasional infections are reported in orangutans (Mul *et al*., 2007), *Colobus* spp. (Gillespie *et al*., 2005), and other non-human primates, such as red guenon (*Cercopithecus ascanius*; Gillespie *et al*., 2004), baboons (Kooriyama *et al*., 2012), Verreaux's sifaka (*Propithecus verreauxi;* Rasambainarivo *et al*., 2014) and blue monkeys (*Cercopithecus mitis*; Appleton *et al*., 1994).

Muhangi *et al*. (2021) reported anoplocephalid cestodes in about a third of 60 mountain gorillas necropsied between 1985 and 2007 in both Virunga and Bwindi and even though simultaneous presence of cestodes and haemorrhagic and/or hyperaemic mucosa of the small intestine (8/60 individuals) and villous blunting (2/60 individuals) were detected, no lesions were observed in 14 animals with cestodiasis. Moreover, during necropsies performed on mountain gorilla individuals deceased e.g., from trauma and generally in good condition, large number of tapeworms were found in the intestines (GD, *personal observation*). It appears that unlike *A. perfoliata,* which is strongly associated with colic in horses (Mezerova *et al*., 2007; Back *et al*., 2013), *A. gorillae* does not have significantly negative health effects on its host.

In all anoplocephalid cestodes, gravid proglottids detach from an adult (Redmond, 1983; Pafčo, 2018) and are passed through the intestines with the content. Based on the species, proglottids can be shed intact, or disintegrate during their way through the gastrointestinal tract releasing the eggs. Redmond (1983) reported that *A. gorillae* eggs remain in proglottids, however, we did not observe any eggs in any of the 5 proglottids examined. The almost ubiquitous presence of *A. gorillae* eggs in feces suggests that the eggs are released from the proglottids into the intestinal content, similarly to some other anoplocephalid species (Schuster, 1991; Deplazes *et al*., 2016). As a result, coproscopy is a sufficient method for the detection of cestodes in mountain gorillas unlike *A. perfoliata* in horses (Traversa *et al*., 2008; Tomczuk *et al*., 2014). Traditionally, the egg quantities (expressed as EPG) are used as a proxy of infection intensity in helminths parasitizing both domestic animals and wildlife (Williamson *et al*., 1998; Chapman *et al*., 2006; Tomczuk *et al*., 2014; Burgunder *et al*., 2017). Individuals with high EPG would expectedly be at higher risk of clinical disease and, due to an uneven distribution of macroparasites commonly found within host populations (Shaw *et al*., 1998; Wilson *et al*., 2001), be also the main reservoir and source of infection for other individuals when shedding most eggs to the environment. From the population management point of view, such animals would be the best targets for interventions such as anthelminthic treatment (Nielsen *et al*., 2019). Ashford *et al*. (1996) reported that mountain gorilla silverbacks in Bwindi shed most cestode eggs, while infants shed no eggs or only a low number of eggs. In our previous study (Petrželková *et al*., 2021), infants in the Virunga population also shed significantly fewer eggs than all other age categories, but that was the only significant trend applicable at the population level. In certain groups, a peak of infection was detected between 10 and 20 years of age and similarly, relationship between group size and egg counts was observed in some groups and/or areas (Petrželková *et al*., 2021). Closer inspection of *A. gorillae* egg shedding patterns in this study revealed a significant EPG fluctuation in individual mountain gorillas within short time periods. Even animals with extreme EPG values (defined in our case as more than 1000 EPG) usually had only 1 sample with such high EPG value. Among 8 individuals with repeatedly high EPG, all age categories as well as both sexes were represented, suggesting that there is no obvious trend regarding the sex or age, but as the number of such individuals was low, we did not test the hypothesis by statistical methods. Egg shedding fluctuation greatly complicates using EPG as a proxy for infection intensity in *A. gorillae*, and, if only a single value is considered for an individual, it may be largely misleading.

Wildlife parasitology, especially in cases of endangered and/or elusive species, relies on noninvasively collected samples. In our case, the *A. gorillae* prevalence in unidentified nest fecal samples and in samples from identified individuals from the same group showed comparable results, which has implication for the gorillas' health monitoring. One-time examination of easily accessible unidentified nest samples provides reliable information about helminth prevalence in the given group. However, multiple sampling of individuals confirmed that even though the cestodes commonly occur in mountain gorillas, the number of gorilla individuals detected as *A. gorillae*-positive further increases with the number of samples tested. It has been suggested previously that at least 3 samples should be examined from an individual primate to reliably detect the spectrum of parasites infecting given individual and to determine realistically the parasite prevalence in a primate group or population (Huffman *et al*., 1997; Muehlenbein, 2005). Our results confirm that such a minimum of samples is needed also for assessment of a parasite as prevalent as *A. gorillae*.

In conclusion, the adult cestodes collected during necropsies helped us establish the identity of *A. gorillae* and provide reference sequences essential for phylogenetic analyses as well as for the development of advanced diagnostic tools. We were unable to associate observed high abundance of *Anoplocephala* with any pathologies, suggesting that the anoplocephalid infection does not represent a threat for the studied subpopulation of *G. beringei beringei*.

## Supporting information

Červená et al. supplementary material 1 Červená et al. supplementary material

Červená et al. supplementary material 2 Červená et al. supplementary material

Červená et al. supplementary material 3 Červená et al. supplementary material

Červená et al. supplementary material 4 Červená et al. supplementary material

Červená et al. supplementary material 5 Červená et al. supplementary material

Červená et al. supplementary material 6 Červená et al. supplementary material

Červená et al. supplementary material 7 Červená et al. supplementary material

## Data Availability

All sequences produced in this study are deposited in the GenBank under accession numbers OR863079-91, OR863669-80 and OR864671-76. A collection of *A. gorillae* adult specimens used in this study was deposited to the Museum of Environment, Karongi District, Rwanda. Contact: fdushimana@rwandaheritage.gov.rw

## Supplementary material

The supplementary material for this article can be found at https://doi.org/10.1017/S0031182023001178
