# Supplementary material for: Anoplocephalid tapeworms in mountain gorillas (Gorilla beringei beringei) inhabiting the Volcanoes National Park, Rwanda
Source: Parasitology. 2023 Nov 29;151(2):135–50. doi: 10.1017/S0031182023001178 (PMC10941052; doi:10.1017/S0031182023001178)
Supplement: Červená et al. supplementary material 6 — Červená et al. supplementary material [file S0031182023001178sup006.docx]

Table S1. PCR assays. Cycling conditions for all PCRs used in this publication. Reaction mix consisted of TopBio 2× MasterMix (TopBio, Vestec, Czechia), 0.8 μM of each primer, and 2.0 μl of extracted DNA in a total volume of 25 μl. In the 2^nd^ round of the nested PCR assay, 1 μl of PCR product from the 1st round was used as a template.

| Assay | PCR round | Primer ID | Primer sequence (5´-3´) | Amplicon (bp) | Cycling conditions | Reference |
| --- | --- | --- | --- | --- | --- | --- |
| diagnostic PCR for *Anoplocephala* (ITS2) | 1st | S18 | TAA CAG GTC TGT GAT GCC | ~1 100 | initial denaturation 3´ at 96°C, 35× 15´´ at 95°C, 15´´ at 55°C, 30´´ at 72°C and final extension 12´at 72°C. | Jousson et al. 1999 |
|  |  | L3T | CAA CTT TCC CTA CGG TAC TTG |  |  | Jousson et al. 1999 |
|  | 2nd | AP-ITS2_3F | AAT TGT GGG GGC TTC TCT TA | ~240 | initial denaturation 3´ at 96°C, 35× 15´´ at 95°C, 15´´ at 55°C, 9´´ at 72°C and final extension 5´at 72°C. | Drogemueller, 2004 |
|  |  | AP-ITS2_2R | ATA AAG AAA GGC ACG AGG T |  |  | Drogemueller, 2004 |
| ITS1 - *Bertiella* specific |  | 201_F | TAT TGC CTA CCT TCG GTG G | ~300 | initial denaturation 3´ at 96°C, 35× 15´´ at 95°C, 15´´ at 60°C, 7´´ at 72°C and final extension 2´at 72°C. | this study |
|  |  | 520_R | TGT AAT AGA ACT CGA CGC ATA G |  |  |  |
| 18S SSU (Anoplocephalids) |  | BF | GGA CAC TAT GAG GAT TGA CAG A | ~600 | initial denaturation 3´ at 96°C, 35× 15´´ at 95°C, 15´´ at 55°C (optionally 52°C), 10´´ at 72°C and final extension 5´at 72°C. | Doležalová et al. 2015 |
|  |  | 18S_2445R | TTG GTC GTC TTC TCA GCA |  |  | this study |
| ITS1 (Anoplocephalids) |  | BertITS1_F | CTG CGG AAG GAT CAT TAC AC | ~600 | initial denaturation 3´ at 96°C, 35× 15´´ at 95°C, 15´´ at 55°C (optionally 52°C), 10´´ at 72°C and final extension 5´at 72°C. | McLennan 2017 |
|  |  | BertITS1_R2 | GCA GTC TGC GAT TCA CAT TA |  |  | McLennan 2017 |

References:

Jousson, O, Bartoli, P and Pawlowski, J (1999). Molecular identification of developmental stages in Opecoelidae (Digenea). *International Journal for Parasitology* **29**, 1853-1858. doi: 10.1016/s0020-7519(99)00124-1

Drogemuller, M, Beelitz, P, Pfister, K, Schnieder, T and von Samson-Himmelstjerna, G (2004) Amplification of ribosomal DNA of Anoplocephalidae: Anoplocephala perfoliata diagnosis by PCR as a pssible alternative to coprological methods. Veterinary Parasitology 124, 205-215. doi: 10.1016/j.vetpar.2004.07.012.

Doležalová, J, Vallo, P, Petrželková, KJ, Foitová, I, Nurcahyo, W, Mudakikwa, A, Hashimoto, C, Jirků, M, Lukeš, J, Scholz, T et al. (2015) Molecular phylogeny of anoplocephalid tapeworms (Cestoda: Anoplocephalidae) infecting humans and non-human primates. Parasitology 142, 1278-1289. doi: 10.1017/S003118201500058X.

McLennan, MR, Hasegawa, H, Bardi, M and Huffman, MA (2017) Gastrointestinal parasite infections and self-medication in wild chimpanzees surviving in degraded forest fragments within an agricultural landscape mosaic in Uganda. PLOS ONE 12, e0180431. doi: 10.1371/journal.pone.0180431
