# Supplementary material for: Anoplocephalid tapeworms in mountain gorillas (Gorilla beringei beringei) inhabiting the Volcanoes National Park, Rwanda
Source: Parasitology. 2023 Nov 29;151(2):135–50. doi: 10.1017/S0031182023001178 (PMC10941052; doi:10.1017/S0031182023001178)
Supplement: Červená et al. supplementary material 7 — Červená et al. supplementary material [file S0031182023001178sup007.docx]

**Table ST2.** Overview of samples evaluated as cestode-negative by Mini-FLOTAC^©^ which contained cestode eggs (detected by sedimentation technique) and/or cestode DNA detected either by *Anoplocephala*-specific assay or *Bertiella-*specific assay. + marks a positive sample, - marks a negative sample. Sequences were obtained from all PCR positive samples.

| sample ID | Group | EPG sediment | PCR Anop | PCR Bert |
| --- | --- | --- | --- | --- |
| CZ54 | Pablo | 0 | + | - |
| CZ68 | Sabyinyo | 70 | + | - |
| CZ97 | Hirwa | 10 | + | - |
| CZ115 | Isimbi | 0 | + | - |
| CZ181 | Kwitonda | 29 | + | - |
| CZ461 | Isabukuru | 2139 | + | - |
| CZ472 | Pablo | 173 | + | - |
| CZ477 | Urugwiro | 16 | + | - |
| CZ487 | Ntambara | 0 | + | - |
| CZ556 | Kwitonda | 6 | + | - |
| CZ607 | Kureba | 20 | + | - |
| CZ629 | Hirwa | 78 | - | + |
| CZ635 | Titus | 5 | - | - |
| CZ652 | Musilikale | 56 | + | - |
| CZ655 | Hirwa | 1262 | - | + |
| CZ706 | Hirwa | 0 | + | - |
| CZ873 | Amahoro | 0 | + | - |
| CZ968 | Susa | 17 | + | - |
| CZ1086 | Musilikale | 30 | + | - |
